# Supplementary figures and images for: The Effects of Agave Fructans in a Functional Food Consumed by Patients with Irritable Bowel Syndrome with Constipation: A Randomized, Double-Blind, Placebo-Controlled Trial
Source: Nutrients. 2023 Aug 10;15(16):3526. doi: 10.3390/nu15163526 (PMC10460012; doi:10.3390/nu15163526)

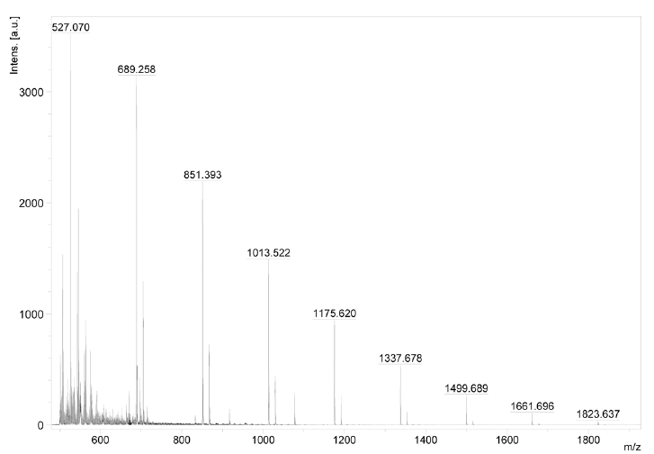

Supplement: Supplementary file 1 [file nutrients-15-03526-s001.zip › nutrients-2524524-supplementary-Figure S1.png]
